# Assessing the Influence of Visual-Taste Congruency on Perceived Sweetness and Product Liking in Immersive VR

**DOI:** 10.3390/foods9040465

**Published:** 2020-04-09

**Authors:** Yang Chen, Arya Xinran Huang, Ilona Faber, Guido Makransky, Federico J. A. Perez-Cueto

**Affiliations:** 1 Department of Food Science, University of Copenhagen, Rolighedsvej 26, 1958 Federiksberg C, Denmark; yangchen@food.ku.dk (Y.C.); ilona.faber@food.ku.dk (I.F.); 2 School of Design, Royal College of Art, Kensington Gore, London SW7 2EU, UK; xinran.huang@network.rca.ac.uk; 3 Department of Psychology, University of Copenhagen, Oester Farimagsgade 2A, 1353 Copenhagen K, Denmark; gm@psy.ku.dk

**Keywords:** sweetness perception, visual cues, virtual reality, frontal alpha asymmetry

## Abstract

This study was designed to assess whether the combined effect of taste-congruent and incongruent extrinsic visual cues presented in virtual reality (VR) influences the perception of sweetness and product liking. Three VR environments (sweet-congruent, sweet-incongruent, and neutral) were created based on the evidence in existing literature. Participants tasted the same beverage in three VR environments and evaluated the environment and beverage liking, as well as perceived taste intensity (sweetness, sourness, and bitterness), congruency, comfort, and environment vividness. Frontal EEG alpha asymmetry (FAA) was also recorded as a complementary physiological measurement of overall liking. The results showed that the perceived sweetness of the beverage was significantly elevated in a sweet-congruent environment versus the other environments. Visual-taste congruency did not seem to have an effect on beverage liking and overall liking, whereas an increase in environment liking was found in the incongruent environment versus the other environments. These findings confirmed the significant influence of taste-specific visual cues on flavour perception, while the successful use of VR in the study provided insight into future applications of taste-specific VR environment in the modulation of flavour perception and sugar reduction.

## 1. Introduction

Our senses are continuously stimulated while we eat, and our brain filters and organises sensory information into an ultimate perception of flavour. Vision, as one of the five basic senses, which also contributes to the formation of the final flavour perception [1]. A growing body of research shows that visual cues, both intrinsic and extrinsic, are able to influence acceptance and perceived flavour [2,3,4,5,6]. At the same time, congruency of different sensory inputs has also been shown to increase our recognition speed [7], as well as product liking [8,9,10,11,12,13,14]. In this study, using both subjective and physiological measurements, we focus on the influence of combined visual cues on the perception of sweetness, as well as product liking.

### 1.1. Influence of Visual Cues on Perception of Sweetness

Visual factors such as shape, colour, and visual texture have been shown to affect perceived intensity of sweetness. Roundness is usually associated with sweetness while angular shapes are associated with bitterness, saltiness, and sourness [3,5,15,16,17,18]. For instance, round plates and food representation have increased sweetness ratings by 17% [3] while round-shaped chocolates are rated 30% sweeter than angular-shaped chocolates [5]. Similar associations were also found for packaging typeface [19], packaging shape [20], and face shape [21]. Apart from shape, colour also has a role in sweetness perception. Intrinsic colour cues such as red/pink colouring have been found to increase the intensity of sweetness while black is linked to bitterness [4,22,23]. Similar effects have been observed with extrinsic colour cues such as the colour of the container [2] and colours in a dining environment [24]. Such an effect is coherent with evidence on people who systematically associate colours with specific tastes [17,25,26,27].

However, some studies posit that it is the contrast between food colour and background colour that changes perceived sweetness [28,29,30]. For example, a pink strawberry mousse was rated 15% sweeter when placed on a white plate than when placed on a black plate [29]. Moreover, visual texture also plays a role in taste perception [31], and surface texture has long been shown to influence taste and hedonic evaluation [32,33,34,35]. Biscuits have been rated sweeter when served on a smooth and shiny plate than when served on a rough and grainy plate [33]. Furthermore, angular surface patterns have enhanced perceived bitterness as compared with round surface patterns [32]. Although evidence regarding the influence of visual texture on flavour is relatively scarce, it could potentially be similar to the evidence from the aforementioned studies on the influence of tactile feedback on flavour perception. In the current study, we adapted evidence gathered in the literature to investigate how combined visual cues in VR environments influence perceived intensity of sweetness, as well as beverage liking.

### 1.2. Application of VR in Sensory and Consumer Research

With advances in immersive technologies such as mixed VR, augmented reality (AR), and mixed reality, increasing numbers of researchers have started to explore applications of these technologies in sensory and consumer studies [36,37,38,39]. Although conducting studies in natural and real-life settings tends to increase test validity and consumer engagement, it can be tricky and costly [40,41,42]. VR is able to create a sense of immersion that evokes feelings of presence and involvement which in turn make VR a cost-effective alternative to conducting studies in real-life settings [43]. Additionally, contextual factors can be more easily standardised in these immersive environments as compared to natural settings [44,45]. Beyond such applications in research, immersive technology is developing at an extraordinary speed in its commercial use. Total spending on AR and VR products and services will rocket, according to a compound annual growth rate of 113.2% projected for the period from 2017 to 2021 [46]. This rapid growth of immersive technology means that research insights acquired today can be readily adapted and utilised in people’s homes and businesses to empower consumer experiences and to change behaviours. For these reasons, we chose VR as a medium capable of presenting all visual cues to the participants in our experiment.

### 1.3. Objective Biometric Measurement of Liking

Conventional sensory and consumer trials usually rely on subjective self-reported responses and the measurement of product liking (i.e., hedonic rating) is mainly based on intrinsically individual differences in perception and liking [47]. It is helpful to use a second type of measurement to provide further evidence. Nowadays, there is a growing interest in applying biometric measurements such as eye tracking, electroencephalography (EEG), and facial recognition in sensory and consumer studies, therefore, adding an objective complement to measures of sensory perception [39,48,49,50,51,52,53,54,55,56]. Among these biometric instruments, EEG is a common non-invasive neuroimaging technique that records electrical activity along the scalp. It records voltage fluctuations caused by ionic current flow among neurons, reflecting brain responses during different tasks [57]. The EEG signal spectral power difference in the alpha frequency band (8 to 13 Hz) between the left and right prefrontal cortex, also known as frontal alpha asymmetry (FAA), is a neurophysiological correlate to the positive effect [58,59]. According to the approach-withdrawal model [60], right-frontal cortical regions facilitate withdrawal motivation, while the left-frontal cortical regions mediate approach motivation [61,62]. This means that individuals with greater right-frontal cortical activation tend to exhibit withdrawal-related behaviour while individuals with greater left frontal cortical activation exhibit approach-related behaviour [59,61]. Therefore, it is possible to use the asymmetry index as an indicator of the appreciation/avoidance tendency. In sensory and consumer studies, FAA was used to study perceived pleasantness towards sensory stimulus [63,64]. In this study, we recorded EEG data for participants drinking a sweet beverage in ”sweet”, ”bitter”, and ”neutral” VR environments. We compared the FAA at electrodes F3 and F4 during tastings in three different VR environments to assess individuals’ affinity complementary to self-reported hedonic ratings obtained in a survey.

The main focus of this study is to investigate individual perception of sweetness in virtual environments simulating visual-taste congruency and incongruency. The first aim of the study is to assess whether a visual-taste congruent environment can lead to increased perceived intensity in sweetness. The second aim is to investigate whether congruent environments increase product liking. On the basis of previous studies, our hypothesis was that perceived intensity of sweetness, as well as beverage liking, would be greater in a sweet-congruent environment than in an incongruent or neutral environment.

## 2. Materials and Methods

A total of 41 participants (17 males and 24 females, mean age 27.73 ± 2.12 years) were recruited using both direct and online recruitment procedures in Denmark. The selection criteria were as follows: right-handed healthy individuals with normal or corrected-to-normal vision, no history of neurological or psychiatric disorders and no synaesthesia. The current study adhered strictly to the Declaration of Helsinki and ethical approval was obtained from the local ethics committee (at University of Copenhagen). All individuals gave written informed consent and a six-digit identification code was assigned to each individual. Data collection was handled according to the General Data Protection Regulation (GDPR).

### 2.1. Experimental Procedures

**Figure 1:**
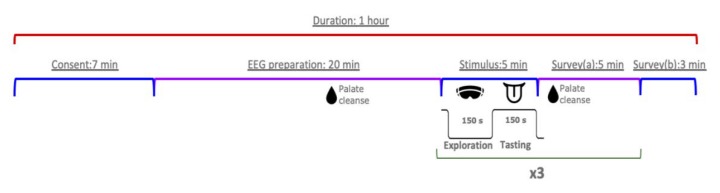
Overall experimental procedures.

The duration of the experiment was about 1 hour in each case; all participants were tested individually (Figure 1). Each was asked to read and sign a consent form immediately upon arrival, and then the researcher briefly explained the experimental procedures, potential risks, and individual rights. They were told that the study investigated consumer preference for three types of berry-flavoured beverages with similar ingredient compositions. Next, participants were (after scalp cleaning procedures) outfitted with an EEG headset. Optimisation of data recording quality was done before a palate cleanse using water to remove any taste residues. Afterwards, participants entered the first VR environment and had the opportunity to freely explore the environment for 150 s (exploration phase). This was followed by a tasting phase, where participants drank one of the test beverages via a plastic straw while being immersed in the VR environment for 150 s and their brain signals were recorded (Figure S1). During the tasting phase, subjects were instructed to sit calmly and avoid unnecessary movements to prevent movement artefacts. A verbal reminder was given by the researcher 30 s before the end of the tasting session. After removing the VR headset, participants filled in a five minute questionnaire regarding the test beverage and environment using a pen while a palate cleanse was performed (Survey a). Altogether, the exploration phase, the tasting phase and the questionnaire lasted for 10 min. Next, the 10 min stimulus and test survey phase were repeated twice in a different environment each time. In total, three VR environments were presented to the participants, in a random order generated by an algorithm. At the end of this process, each participant completed a short questionnaire regarding demographic information, self-reported health state, sweetness liking and preference, as well as VR familiarity (Survey b). Each participant, upon completion, was compensated with a gift set, valued at approximately 100 Danish crowns.

### 2.2. Stimulus

#### 2.2.1. Taste Stimuli and Palate Cleanser

All taste stimuli were the same in this study. In each VR environment, participants received 100 mL of test beverage contained in a white plastic cup and consumed the beverage using a plastic straw. Participants were asked to finish drinking the test beverage (100 mL) if possible. They were also given the option to recap the taste of the test beverage before the end of the tasting session. Concentrated grenadine syrup (SAS, Teisseire, France) was diluted with water at a 1:7 ratio based on the producer’s recommendation and a pilot tasting session. Table S1 shows the nutritional content of the taste stimulus. The test beverage was freshly made for each test day under hygienic conditions and mixed thoroughly before administration. The 100 mL amounts, in their plastic cups, were placed in the test room one hour prior to the experiment and the room temperature was kept constant at 22 °C to ensure maximum taste sensitivity in the 20 to 30 °C temperature range [65,66]. Palate cleansing was performed to remove any residue before and between taste samples to minimise carryover effects [67,68,69]. Water, one of the most effective palate cleansers for sweetness [70], was used in this study. Participants rinsed their mouths with water before each tasting in order to re-establish a baseline oral environment.

#### 2.2.2. Visual Stimuli

**Figure 2:**
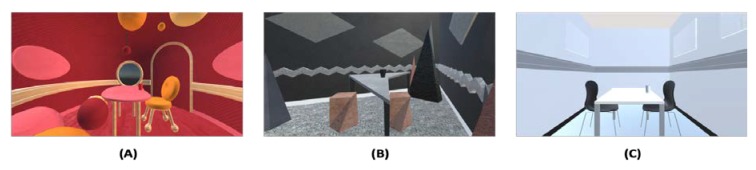
Three VR environments used in the study. (**A**) Sweet environment; (**B**) Bitter environment; and (**C**) Neutral environment.

Three VR environments were used as visual stimuli. According to the existing literature, sweet-congruent visual cues such as round shapes, pink/red-shaded colours, and soft/smooth visual textures were incorporated into the design of the sweet environment. Meanwhile, bitter-congruent visual cues such as angular shapes, black/grey-shaded colours, and rough/grainy textures were incorporated into the incongruent/bitter environment, and the neutral environment was designed based on the test room itself, with a plain white background (Figure 2). Models were built using Rhinoceros three-dimensional (3D) software (Rhino 6.0) and rendered using Unity software (Unity, version 2019.2.13). With slight modifications, all three environments were designed based on the size of the test room (length = 3.6 m, width = 2.7 m, and height = 2.55 m), while the furniture and decorations in each environment were also adjusted for similarity in size, scale, and spatial arrangement. Android application packages (APK) of the VR environments were made freely available through three QR codes (Figure S2). Participants experienced the VR contexts through a Samsung Gear VR headset powered by Samsung Galaxy S7 smartphone. All three VR environmental designs were verified through a pilot questionnaire to ensure visual-taste association.

### 2.3. Questionnaires

#### 2.3.1. Pilot Questionnaires

The taste association of all the visual stimuli was verified via an online questionnaire (Survey monkey) with a total number of 138 participants (69 males and 69 females, mean age 34.20 ± 13.82 years). Participants were asked to associate three designed environments (in two-dimensional (2D) format) with sweetness, bitterness, sourness, and saltiness. They also rated the liking of three environments using a nine-point hedonic scale.

#### 2.3.2. Experiment Questionnaires

Two questionnaires were given to the participants during the experiment. The first questionnaire (for survey a) was given immediately following every tasting session, using a nine-point scale with −4 = “not at all”, 0 = “neutral”, and +4 = “extremely”. We measured the levels of comfort, taste intensity (sweetness, sourness and bitterness), beverage and environment liking, visual-taste congruence, and environment vividness (Table S4). The second questionnaire (for Survey b) was given at the end of the experiment, capturing demographic information, self-reported health state, exercise frequency, sweet food consumption frequency, sweet food liking, and VR familiarity (Table S5). Self-reported states of health, along with frequencies for exercise and sweet food consumption, were registered on a five-point scale, whereas sweet food liking and VR familiarity were measured on a seven-point scale.

### 2.4. EEG Recording

Neurophysiological data was acquired using a wireless nine-channel headset (ABM B-Alert X10) with a sampling rate of 256 Hz and electrodes positioned (in reference to both left and right mastoids) at POz, Fz, Cz, F3, F4, C3, C4, P3, and P4. The headset was connected via Bluetooth to a Windows 10 computer. Data collection was carried out using the iMotion research software platform. Channel impedance was checked throughout the experiment (X-Series Basic, version 3.2.5.0) to ensure good impedance, and only those individual experiments which yielded valid data for electrodes F3 and F4 were accepted as input for this study. Three sets of data were excluded from the study due to bad signal-to-noise ratio.

Offline data processing was performed using a Matlab EEGLAB toolbox [71]. Baseline correction was conducted throughout the recordings to reduce potential electrode drift. Excessive noises, with amplitudes below or above 150 μV, were excluded. Independent component analysis (ICA) was performed to remove ocular artefacts using the EEGLAB toolbox. FAA was assessed based on published recommendations [61,72]. In brief, power values in the alpha range (8–13 Hz) were extracted from the F3 and F4 channels. An asymmetry score was calculated by subtracting the F4 values from the F3 values and dividing by the sum for the two electrodes (formula below). One thing to be noted is that there is an inverse relationship between alpha power and cortical activity, which means decreased alpha power reflects increased regional cortical activity [72]. Therefore, a more negative FAA value would indicate higher activation of the left frontal regions (greater liking).

(1) $$\frac{\log\left(F3\right)-\log\left(F4\right)}{\log\left(F3\right)+\log\left(F4\right)}$$

### 2.5. Data Analysis

We used SPSS (version 24, IBM Corp, Armank, NY, USA) for statistical analysis. A one-way analysis of variance (ANOVA) was followed by a post hoc by Tukey’s test, conducted to assess differences in perceived taste (sweetness, bitterness, and sourness), beverage and environment liking, visual-taste congruency, comfort, and environment vividness, as well as the difference in FAA index (objective overall liking measure) in three different settings. Pearson correlation coefficients were used to assess interrelations for congruency and liking (beverage, environment, and overall liking). Gender difference in all the liking attributes was interpreted using the Mann–Whitney U test. All *p*-values below 0.05 were considered statistically significant.

## 3. Results

### 3.1. Visual-Taste Association and Liking of Three VR Environments (Pilot Survey)

**Figure 3:**
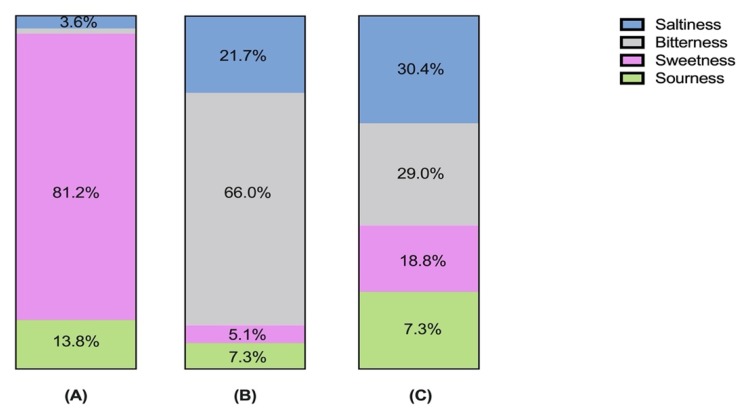
Association between four tastes and (**A**) sweet environment; (**B**) bitter environment; and (**C**) neutral environment (in terms of %).

The association between tastes and designed environment and liking was investigated in a pilot survey (*n* = 138). The sweet environment was 81.2% associated with sweetness while the bitter environment was mainly associated with bitterness (66.0%) and least associated with sweetness (5.1%). The neutral environment had a relatively balanced association with four different tastes (Figure 3). This result confirms the designed VR environments as sweet-congruent, bitter-congruent, and neutral. The average liking of sweet, bitter, and neutral environments was 4.8 (SD = 1.7), 4.4 (SD = 2.1), and 4.4 (SD = 1.5), respectively (Table S3). No significant difference was observed.

### 3.2. Demographic Characteristics and Preferences

Demographic characteristics and preferences of participants are shown in Table S2. The average familiarity with VR headsets was 4.7/1.3 (mean/SD), corresponding to “familiarity”. The male participants were found to be more familiar with VR headsets than the females (*p* < 0.05). The average self-rated health status was “good” (4.4/0.5, mean/SD) while the average exercise frequency was “a few times per week” (3.7/0.8, mean/SD). Average consumption frequency of sweet food/beverage was “a few times per week” (3.5/0.9, mean/SD) while the average liking of sweet foods/beverages was “like slightly” (4.7/1.2, mean/SD).

### 3.3. Visual-Taste Congruency on Perceived Taste

**Figure 4:**
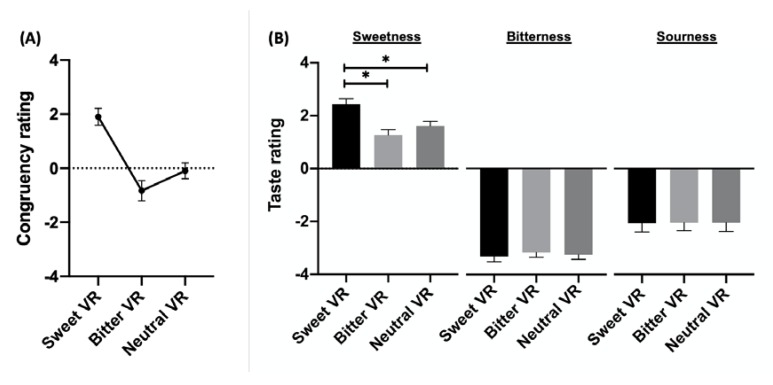
Participants’ rating on (**A**) visual-taste congruency and (**B**) intensity of sweetness, sourness, and bitterness in three VR environments (mean ± SEM). * indicates *p* < 0.05.

Figure 4 illustrates the rating of visual-taste congruency, as well as the influence of each visual environment (sweet, bitter, and neutral) on sweetness intensity. The rating of congruency was the highest in the sweet VR environment (1.9/0.3, mean/SEM) while that in the bitter environment was the lowest (−0.83/0.4, mean/SEM). The rating for sweetness was the greatest in the sweet-congruent environment (2.4/0.2, mean/SEM) while it was the lowest in the incongruent environment (1.3/0.2, mean/SEM). ANOVA analysis also revealed significant effects of the visual environment on the intensity of perceived sweetness (F (2, 120) = 9.391, *p* = 0.0002) with an effect size (η^2^ = 0.135) classified as large (Table 1) [60]. Consistent with the above results, the post hoc Tukey’s test on congruency and perceived sweetness ratings showed that the sweet environment had yielded significantly higher ratings than the bitter environment (Table 2). There was no observed effect on perceived bitterness or sourness.

Environments that do not share a letter are significantly different from each other (*p* < 0.05) in the post hoc Tukey tests.

### 3.4. Visual-Taste Congruency on Liking

To obtain an overview of the interrelations among visual-taste congruency and all the liking responses, Pearson correlation coefficients were generated for congruency and three liking variants in the three different VR environments (Table 3). A positive correlation between congruency on liking and environment liking was observed in all three VR environments, but not for beverage liking or overall liking. Furthermore, a positive correlation was found between environment liking and beverage liking in all three environments. As expected, FAA was negatively correlated to the other three variants, because FAA showed more negative values to indicate higher liking. However, no significance was found. To assess the difference of liking level among the three environments, the ANOVA analysis showed a significant effect of visual environment on environment liking (F (2, 120) = 6.490, *p* = 0.0021, and η^2^ = 0.043). However, no effect was observed in beverage liking and overall liking (FAA).

### 3.5. Gender Difference on Liking

Although no significant difference was observed in environment liking using our pilot questionnaire, a significant difference was shown in environment liking in the experiment questionnaire. Additionally, the gender ratio in the experiment was relatively imbalanced as compared with that of the pilot survey. We suspected that there could be a gender-influenced difference in preferences, therefore, we investigated gender differences in the environment, beverage, and overall liking using the Mann–Whitney U test (Table 4). Gender difference did not appear in the hedonic evaluation or the sweetness rating, across all three environments (*p* > 0.10).

## 4. Discussion

Overall, the results of the study reveal that visual-taste congruency has a significant influence on the perceived intensity of sweetness. The same beverage was rated significantly sweeter in a sweet-congruent environment than in an incongruent (bitter) or neutral environment. A maximum 13% increase in sweetness perception (1.15 points) on a nine-point scale was revealed when participants were immersed in a sweet-congruent VR environment. This result is consistent with a growing body of literature on visual sweetness enhancement [2,3,5,24,73]. With the global obesity crisis closely related to excessive sugar consumption [74,75,76], it is important to build psychological models that enhance individuals’ sweetness perception to help reduce sugar levels in foods and beverages.

Unexpectedly, we did not observe any influence of visual-taste congruency on product liking. In theory, however, mixed results have been shown in the existing literature on the influence of taste-specific visual cues on flavour perception. Some studies have indicated a significant influence of taste-congruent visual cues on product liking [2,14,24] while other studies have not reported this result [5,77]. Additionally, a recent study showed the existence of crossmodal correspondences between physical warmth and light colours [78]. This type of temperature-visual congruency increases consumer preferences only for light-coloured goods under comfortable conditions, which indicates that the level of comfort could play a role in the influence of congruency on product liking. Reflecting on this issue, it is possible that the VR environments presented in the current study were not comfortable enough to increase product liking, as the average comfort rating for the three environments lies was either “slightly comfortable” or “moderately comfortable”. Interestingly, significant results have been shown in environment liking but not found in pilot testing where respondents rated 2D pictures of three VR environments. A maximum 12% increase in environment liking (1.1 points) on a nine-point scale was reported when immersed in a congruent VR environment. We initially suspected it was gender difference that caused this shift of liking, since some of the female participants explicitly reported extreme liking of the sweet environment and the gender ratio was less balanced in the experiment population as compared with that of the pilot population. However, this factor was later eliminated. As a positive correlation was found between environment liking and beverage liking, it seemingly indicates a complementary effect between taste and visual cues on consumer preferences. It is also possible that the 3D VR experience is more real and attractive than 2D images, meaning that the participants like it better through stronger overall affect. An increase of 13% in overall environment liking (1.13 point) was observed after switching to the same nine-point scale. No significant difference was observed in overall liking (FAA), since the results of beverage affinity counteracted the results of environment affinity.

This is the first time that combined taste-specific visual cues were shown to influence sweetness perception in a VR environment. As VR and other immersive technologies become less expensive and easier to use, they can be used as a flexible interface to change eating environments anywhere and anytime. Thus, they could reduce people’s sugar intake by enhancing sweetness perception without compromising the overall taste experience. The results from the current study provide insight into the future design of taste-congruent VR environments. In future, it would be interesting to see if the addition of other sensory-congruent cues (e.g., auditory, tactile, and olfactory) could further modulate individual flavour perception in immersive environments. For example, taste-specific speech sounds [79] and tactile cues [80] can be easily incorporated into immersive environments to influence flavour perception.

The present study has several limitations stemming from the fact that the EEG was recorded while participants tasted the beverage in the VR environment. This means that the resulting FAA values provided an overall indication of the approach/withdrawal tendency towards the tested product and environment, but was unable to specifically represent a response towards environment or beverage. It is possible to obtain such values by recording brain activity separately while being in a VR environment only, or during tasting only. However, this is difficult to fulfil due to logistic reasons. Moreover, the VR environment could be more interactive and real with motion sensors added to the setup. For example, participants could reach for the cup holding the beverage as represented in the environment instead of blindly receiving the beverage from the researcher. This would require more advanced apparatus to power the software as the current equipment (Samsung gear VR powered by Samsung S7) was unable to do so.

## 5. Conclusions

The current study found that visual-taste congruency could exert a significant influence on an individual’s perception of sweetness but not on product liking. This result provides insight into the design of future taste-congruent environments that could enhance certain flavour perceptions and ultimately change people’s eating behaviour. Immersive technology such as VR could be a useful medium to deliver the above designs in a flexible and cost-effective manner.

## Figures and Tables

**Table 1:** Effect of visual-taste congruency on perceived tastes, liking, congruency, comfort, and environment vividness in terms of degrees of freedom, F value, *p*-value, and effect size.

	*df*	F	*p*-Value	η^2^
**Sweetness**	**2**	**9.391**	**<0.0005**	**0.135**
Bitterness	2	0.155	0.857	0.003
Sourness	2	0.002	0.998	<0.0005
Beverage liking	2	0.563	0.571	0.009
**Environment liking**	**2**	**6.490**	**0.073**	**0.043**
FAA	2	0.563	0.571	0.009
**Congruency**	**2**	**18.530**	**<0.0005**	**0.236**
Vividness	2	2.240	0.111	0.036
Comfort	2	1.041	0.356	0.017

Significant effects (*p* < 0.05) are shown in bold.

**Table 2:** Average ratings with standard deviation and standard error per visual environment.

	ENVIRONMENT	MEAN(SD)	SEM	COMPARISON
SWEETNESS	Sweet	2.42(1.31)	0.21	A
	Bitter	1.27(1.34)	0.21	B
	Neutral	1.61(1.12)	0.17	AB
BITTERNESS	Sweet	−3.32(1.29)	0.2	A
	Bitter	−3.17(1.12)	0.17	A
	Neutral	−3.24(1.16)	0.18	A
SOURNESS	Sweet	−2.07(2.07)	0.32	A
	Bitter	−2.05(1.90)	0.29	A
	Neutral	−2.05(2.12)	0.33	A
BEVERAGE LIKING	Sweet	1.29(1.72)	0.27	A
	Bitter	0.9(1.77)	0.28	A
	Neutral	1.12(1.53)	0.24	A
ENVIRONMENT LIKING	Sweet	1.39 (1.63)	0.25	A
	Bitter	0.29 (1.60)	0.25	B
	Neutral	0.34 (1.44)	0.23	AB
FAA	Sweet	−0.01(0.07)	0.01	A
	Bitter	0.01(0.07)	0.01	A
	Neutral	0.00(0.07)	0.01	A
CONGRUENCY	Sweet	1.90(1.90)	0.31	A
	Bitter	−0.83(2.40)	0.37	B
	Neutral	−0.10(1.89)	0.29	B
VIVIDNESS	Sweet	1.39(1.75)	0.27	A
	Bitter	0.80(1.78)	0.28	A
	Neutral	0.59(1.82)	0.28	A
COMFORT	Sweet	2.39(1.24)	0.19	A
	Bitter	2.07(1.69)	0.27	A
	Neutral	1.93(1.49)	0.23	A

**Table 3:** Pearson correlation coefficients between environment liking, beverage liking, overall liking (FAA), and congruency in (**A**) the sweet environment, (**B**) the bitter environment, and (**C**) the neutral environment.

**(A) Sweet environment**				
	**Congruency**	**Beverage Liking**	**Environment Liking**	**FAA**
Congruency	1.0	0.12	0.47 **	−0.01
Beverage liking	-	1.0	0.30 *	−0.08
Environment liking	-	-	1.0	−0.34
FAA	-	-	-	1.0
**(B) Bitter environment**				
	**Congruency**	**Beverage Liking**	**Environment Liking**	**FAA**
Congruency	1.0	0.25	0.45 **	−0.24
Beverage liking	-	1.0	0.40 *	−0.21
Environment liking	-	-	1.0	−0.1
FAA	-	-	-	1.0
**(C) Neutral environment**				
	**Congruency**	**Beverage Liking**	**Environment Liking**	**FAA**
Congruency	1.0	0.16	0.18 *	−0.14
Beverage liking	-	1.0	0.31 *	−0.38
Environment liking	-	-	1.0	−0.17

* Indicates significance at the 0.05 level and ** indicates significance at the 0.01 level.

**Table 4:** Gender difference between environment liking, beverage liking, and perceived sweetness intensity in (**A**) the sweet environment, (**B**) the bitter environment and (**C**) the neutral environment.

**(A) Sweet environment**			
	**Environment liking**	**Beverage liking**	**Sweetness**
Male	1(−0.5,2)	2(1,2.5)	2(2,4)
Female	2(0,3)	1(0,3)	3(2,3)
Z	−1.515	−0.890	−0.027
P	0.130	0.373	0.978
**(B) Bitter environment**			
	**Environment liking**	**Beverage liking**	**Sweetness**
Male	0(−1,0.5)	1(−0.5,3)	1(1,2)
Female	0(−1,2)	1(0,2)	1.5(1,2)
Z	−0.860	−0.526	−0.125
P	0.390	0.599	0.901
**(C) Neutral environment**			
	**Environment liking**	**Beverage liking**	**Sweetness**
Male	1(−1,2)	2(1,2)	1(−1,2)
Female	0(0,1)	2(1,2.75)	0(0,1)
Z	−0.464	−0.411	−0.464
P	0.642	0.681	0.642

## Supplementary Materials

The following are available online at https://www.mdpi.com/2304-8158/9/4/465/s1, Figure S1: Photo of a participant tasting test beverage during VR exposure, Figure S2: QR codes for APK files of three VR environments, Table S1: Nutritional content of taste stimulus used in the experiment, Table S2: Background information of experiment participants (*n* = 41) depicted as mean (SD) or n, Table S3: Ratings of environment liking (9-point hedonic scale) in pilot survey (*n* = 138) depicted as mean (SD), Table S4: Questions and choices in experiment survey a, Table S5: Questions and choices in experiment survey b.
