# Supplementary material for: Assessing the Influence of Visual-Taste Congruency on Perceived Sweetness and Product Liking in Immersive VR
Source: Foods. 2020 Apr 9;9(4):465. doi: 10.3390/foods9040465 (PMC7230737; doi:10.3390/foods9040465)
Supplement: Supplementary file 1 [file foods-09-00465-s001.pdf]

foods

Supplementary materials

Table S1. Nutritional content of taste stimulus used in the experiment.

Taste stimulus	Nutritional content (per 100g)
Teisseire grenadine syrup	Energy 328 kcal, fat 0 g, saturates 0 g, carbohydrates 82 g and sugars 82 g.

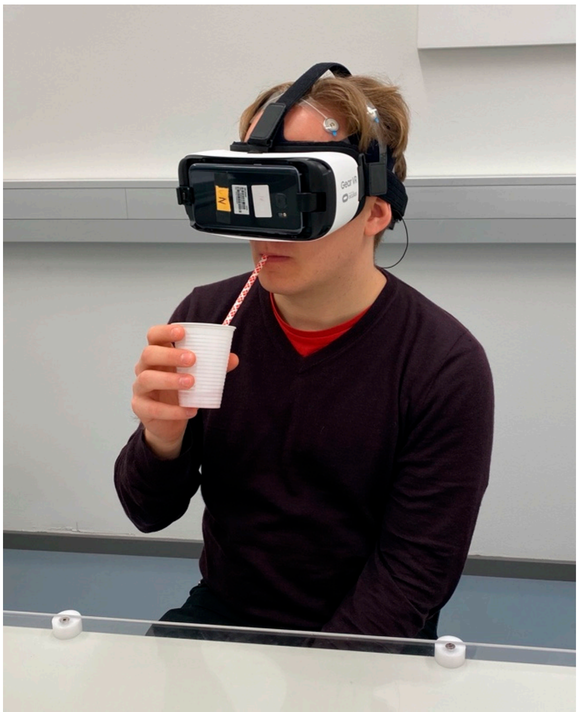

Figure S1. Photo of a participant tasting test beverage during VR exposure.

Table S2. Background information of experiment participants (n = 41) depicted as mean (SD) or n.

	Participants (n=41)
Gender (Male/Female)	17/24
Age	27.7(2.1)
Self-rated health status [1]	4.4(0.5)
Exercise frequency [2]	3.7(0.8)
Sweet food consumption frequency [2]	4(0.9)
Sweet food liking [3]	5(0.2)
Familiarity with VR [4]	5(0.2)

[1]The self-rated health status was measured with a 5-point scale of which 1 = very bad, 3 = neither good or bad; 5 = very good. [2] The frequency of exercise and sweet food consumption were measured with a 5-point scale of which 1 = never, 2 = seldom, 3 = a few times a month, 4=a few times a week,

5=daily. [3] The sweet food liking was measured with a 7-point Likert scale, 1=dislike extremely, 5=neither like or dislike, 7=like extremely. [4] The familiarity level with VR was measured with a 7-point scale, 1= extremely unfamiliar, 5=neither familiar or unfamiliar, 7=extremely familiar.

Table S3. Ratings of environment liking (9-point hedonic scale) in pilot survey (n = 138) depicted as mean (SD).

Environment liking	**Mean (SD)**
Sweetness environment	4.84(1.73)
Bitterness environment	4.38(2.12)
Neutral environment	4.406 (1.45)
All environment	4.54 (1.80)

(A) (B) (C)

Figure S2. QR codes for APK files of three VR environments (A) sweet environment (B) bitter environment (C) neutral environment.

Table S4. Questions and choices in experiment survey a.

<table>
<tr><th>Questions</th><th>Choices</th></tr>
<tr><td>How comfortable are you right now?</td><td rowspan="8">o Not at all. Extremely (uncomfortable/dislike/not sour/not bitter/ not sweet/unmatched/not vivid)
o Very much (uncomfortable/dislike/ not sour/not bitter/ not sweet/unmatched/not vivid)
o Moderately (uncomfortable/dislike/ not sour/not bitter/ not sweet/unmatched/not vivid)
o Slightly (uncomfortable/dislike/ not sour/not bitter/ not sweet/unmatched/not vivid)
o Neutral
o Slightly (comfortable/like it/ sour/ bitter/sweet/matching/ vivid)
o Moderately (comfortable/like it/ sour/bitter/sweet/matching/ vivid)
o Very much (comfortable/like it/ sour/bitter/sweet/matching/ vivid)
o Extremely (comfortable/like it/sour/bitter/sweet/matching/ vivid)</td></tr>
<tr><td>How much did you like the drink you tried just now?</td></tr>
<tr><td>How sour do you think the drink was?</td></tr>
<tr><td>How bitter do you think the drink was?</td></tr>
<tr><td>How sweet do you think the drink was?</td></tr>
<tr><td>How much did you like the VR environment you experienced just now?</td></tr>
<tr><td>To what extent you think the VR environment you experienced just now matches the taste of the beverage you just had?</td></tr>
<tr><td>How vivid do you think the VR environment was?</td></tr>
</table>

Table S5. Questions and choices in experiment survey b.

<table>
<tr><th>Questions</th><th>Choices</th></tr>
<tr><td>Gender</td><td>Male/Female</td></tr>
<tr><td>Age</td><td>Answer in numbers</td></tr>
<tr><td>How would you rate your health status?</td><td>Very Bad/Bad/Neither good or bad /Good/Very Good</td></tr>
<tr><td>How often do you exercise?</td><td rowspan="2">Never/Seldom/A few times per month/A few times per week/Daily</td></tr>
<tr><td>How often do you consume sweet food/beverage?</td></tr>
<tr><td>How much do you like sweet food/beverage in general?</td><td>Dislike very much/ Dislike moderately/ Dislike slightly/Neither like or dislike/Like slightly/ Like moderately/ Like very much</td></tr>
<tr><td>Before this experiment, how familiar you were with virtual reality glasses?</td><td>Very much unfamiliar(never heard of) /Moderately familiar/ Slightly unfamiliar/Neither familiar or unfamiliar/Slight familiar/Moderately familiar/ Very much familiar</td></tr>
</table>
